# A case report on 2 unique presentations of upper extremity deep vein thrombosis

**DOI:** 10.1097/MD.0000000000009944

**Published:** 2018-03-16

**Authors:** Muharrem Yunce, Ashwyn Sharma, Evan Braunstein, Michael B. Streiff, Ying Wei Lum

**Affiliations:** a Department of Medicine, MedStar Franklin Square Medical Center; b Department of Vascular Surgery and Endovascular Therapy; c Department of Medicine Hematology Division, The Johns Hopkins University School of Medicine, Baltimore, MD, USA.

**Keywords:** DVT, management, upper extremity

## Abstract

**Rationale:**

Thoracic outlet syndrome (TOS) is a rare cause of upper extremity deep vein thrombosis (UEDVT). The treatment usually involves catheter directed thrombolysis followed by systemic anticoagulation. Surgical decompression is frequently recommended after anticoagulation for definitive therapy.

**Patient concerns:**

We report two cases of UEDVT secondary to venous TOS with important clinical presentations.

**Diagnoses:**

Venous TOS.

**Interventions:**

One patient was initially treated conservatively but had a recurrent UEDVT. The second patient had a residual stump from a prior rib resection that was causing compression on the subclavian vein, resulting in recurrent venous symptoms.

**Outcomes:**

Both patients achieved significant improvement in their symptoms at 1 year follow-up.

**Lessons:**

UEDVTs can be debilitating, and may limit activities of daily living. Surgical decompression may offer significant improvement in quality of life and symptom relief in such patients.

## 1 Introduction

Upper extremity deep vein thromboses (UEDVTs) are uncommon, accounting for 1% to 4% of all DVT cases. The most common causes of UEDVT are secondary to central venous catheters, pacemakers, or peripherally inserted central catheters. These causes are responsible for up to 50% of all UEDVTs. Venous thoracic outlet syndrome (VTOS) is a cause of primary UEDVTs, representing 20% to 30% of cases.^[1]^ It is estimated that VTOS currently afflicts 1 to 2 per 100,000 persons per year.

This report describes 2 different cases of VTOS. They were initially treated with 2 different interventions, but recurring symptoms necessitated further surgical treatment. These reports serve to highlight the complex treatment algorithm that is used for managing patients with VTOS.

## 2 Case 1

A 28-year-old right handed woman with a past medical history significant for a DVT in her right upper arm presented to the ED with right upper extremity swelling, vein engorgement, and pain. She first experienced similar symptoms in 2013, when she noted edema and fatigue of her right upper extremity after lifting weights. She was diagnosed with a DVT in her right upper extremity and was treated with thrombectomy and discharged on a 6-month course of Lovenox. In addition, she had a complete hypercoagulability workup which was negative. She re-presented at an outside hospital 2 weeks prior to presentation at our hospital with right upper extremity edema and easy fatigability. She was found to have a recurrent DVT and underwent thrombolysis and angioplasty. She was discharged on Xarelto with outpatient follow-up, before presenting with recurrent symptoms yet again at our hospital.

**Figure 1:**
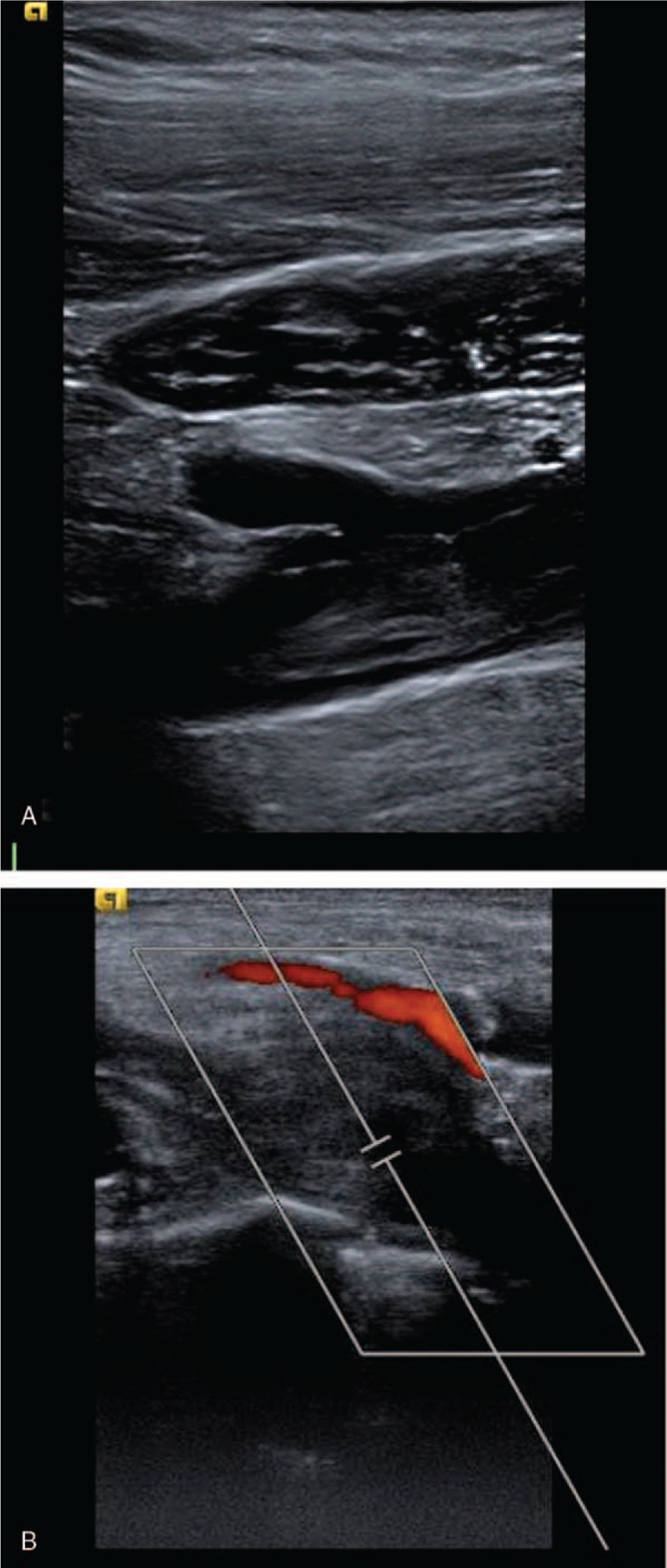
(A, B) Ultrasound duplex demonstrating occlusive right axillary and subclavian venous thrombosis for case 1.

On examination, the patient was noted to have right arm swelling compared to the left, and vein engorgement noted in the entire arm starting from the axillary region. The right arm exhibited increased warmness compared to the left. Venous duplex ultrasound revealed an occlusive thrombus within the basilic, cephalic, and axillary veins that extended into the subclavian vein (Fig. 1). In addition, there was a complete cessation of flow with arm abduction, consistent with the diagnosis of VTOS.

**Figure 2:**
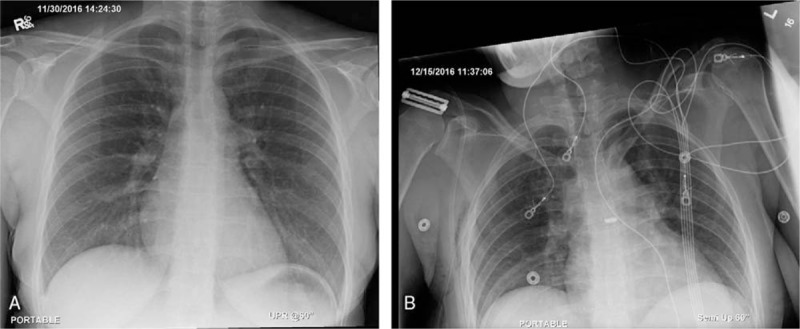
(A, B) Chest X-ray before and after surgery for case 1.

**Figure 3:**
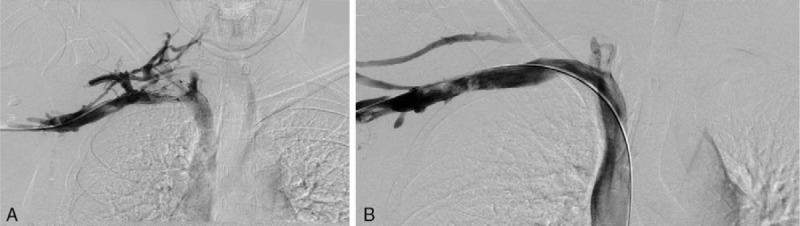
(A, B) Pre- and postangioplasty venogram for case 1.

Given the patient's youth, recent history of heavy upper extremity exercise, physical exam findings, and supportive imaging, a diagnosis of VTOS was made. She underwent a repeat attempt at thrombolysis, but the thrombus was too occlusive to traverse. This was followed by right first rib resection and scalenectomy (Fig. 2). She was discharged on Lovenox following surgery and underwent repeat venogram 2 weeks after surgical decompression, which showed a patent subclavian vein with tight stenosis. Endovascular venoplasty was performed with a 12 mm × 4 cm noncompliant balloon with good technical results, and postballooning angiograms demonstrated a widely patent subclavian vein (Fig. 3). Follow-up venous duplex demonstrated a widely patent subclavian vein. She completed 4 months of Xarelto, currently remains free of symptoms at 1 year, with no recurrence of DVT.

## 3 Case 2

A 53-year-old right handed female with a previous diagnosis of thoracic outlet syndrome (TOS) presented to the ED with right arm swelling and shooting pains of 4 weeks duration. She reported that her first episode of these symptoms occurred 15 years ago. At that time, she presented with right arm swelling, difficulty raising her arm above her head, and pain, and was subsequently diagnosed with an UEDVT. Despite being on anticoagulation, her symptoms persisted, so she underwent a transaxillary right first rib resection with neurolysis and anterior scalenectomy in 2001. She was maintained on anticoagulation for 3 months postoperatively with resolution of her symptoms.

She was asymptomatic for 15 years until July 2016, when she presented with right arm pain, swelling, and difficulty fitting her clothes on the right side. Her symptoms gradually worsened and was seen by a vascular surgeon, who evaluated her and found a narrowing of her subclavian vein. She underwent angioplasty in August with transient improvement of her swelling. She subsequently had a stent placed in the right subclavian vein in September 2016, but her symptoms returned, and she began to notice that her arm was discolored. The patient returned to the vascular surgeon, who performed a computed tomography angiography that showed narrowing and deformity of the stent and a proximal occlusion that was concerning for a clot. She was started on Eliquis in November 2016, but continued to have increased swelling and numbness. She ultimately had a duplex ultrasound which showed a new brachial vein thrombus. She was sent to the ED in for further management.

At our institution, venous duplex ultrasound demonstrated narrowing with the right subclavian vein stent with a partially occlusive thrombus.

**Figure 4:**
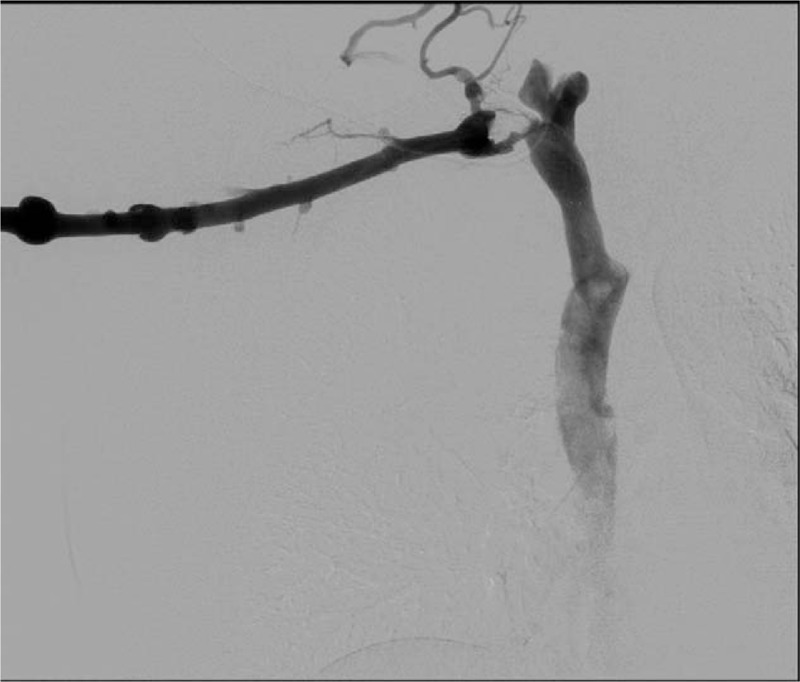
Venogram demonstrating filling defect within the right subclavian venous stent.

**Figure 5:**
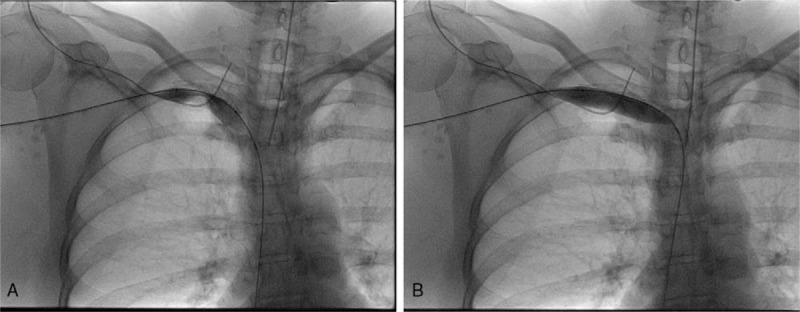
(A, B) Venogram demonstrating inflation of the balloon within the narrowed stent.

**Figure 6:**
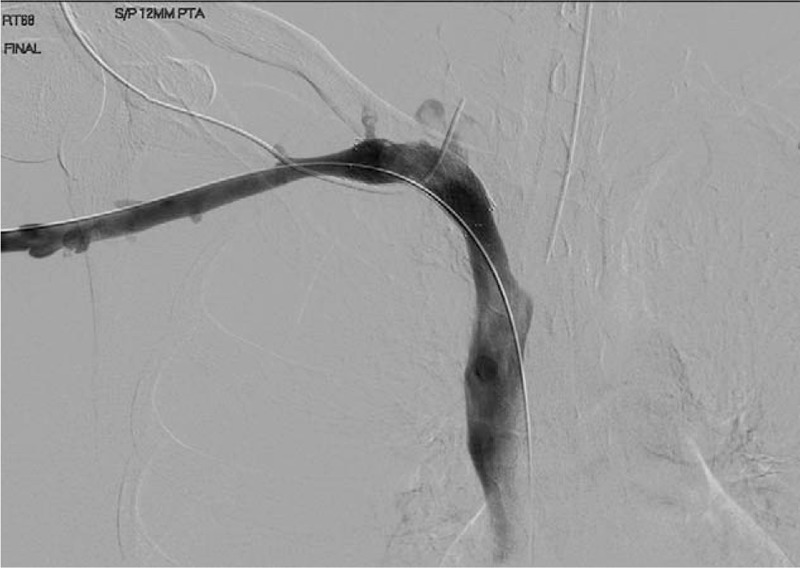
Venogram postangioplasty demonstrating patent right subclavian venous stent.

A computed tomography angiography scan demonstrated persistent compression of the midportion of the right subclavian vein stent by a residual 1st rib remnant on the medial edge adjacent to the sternum. She underwent right infraclavicular residual first rib resection with subsequent endovascular subclavian vein angioplasty with a 12 mm × 4 cm noncompliant balloon (Figs. 4–6). This resulted in re-expansion of the stent. She was discharged on coumadin. On follow-up, her swelling had significantly improved, and on venous duplex ultrasound, the subclavian vein was patent and there was no evidence of a clot. She remains on anticoagulation due to the stent placement with no recurrent episodes of swelling at 1-year follow-up.

## 4 Discussion

UEDVT accounts for 1% to 4% of all DVTs. With the increasing use of central venous catheters, and in particular peripherally inserted central catheters lines, the incidence of UEDVT has been increasing. However, in young, healthy patients who present with UEDVTs, it is important to consider primary causes, such as VTOS. In VTOS, the subclavian vein is compressed inside the costoclavicular space, bounded by the first rib, the clavicle, and the costoclavicular ligament.^[2]^ This may cause scar tissue to form inside of the vein, subsequently decreasing blood flow through the vein. The decreased blood flow allows for the formation of blood clots, causing symptoms for the patient.^[3]^

Patients with VTOS classically present with upper extremity edema with dilated subcutaneous collateral veins over the arm, shoulder, and chest. Patients may also present with cyanosis of the extremity or aching pain with exercise. The right upper extremity is more commonly affected, likely due to the fact that majority of patients are right-handed. Approximately two-thirds of patients with VTOS have a history of vigorous exercise or heavy activity involving the upper extremities.^[4]^ Oftentimes, imaging is necessary to confirm the diagnosis of UEDVT. In these cases, we utilized venous duplex ultrasound. Duplex ultrasound has been recommended as an initial diagnostic tool due to its high sensitivity and specificity. Contrast venography is indicated in patients who have demonstrated pathology on duplex ultrasound and in whom an endovascular intervention is planned.^[3,5]^

The mainstay of treatment is controversial given the lack of randomized trials and the overall rarity of the disease. The treatment for VTOS is historically centered on anticoagulation and symptomatic treatment. However, these therapies are associated with significant disability, recurrent thrombosis, and persistent symptoms.^[6]^ Other authors have suggested that contemporary approaches, which center on catheter-directed thrombolysis followed by a 6-month course of anticoagulation, are more suitable in the acute symptomatic setting.^[7]^ Although these methods have some short-term benefits, it is unfortunately not the definitive treatment in patients with UEDVT. From our experience, early surgical intervention using a transaxillary approach for removal of the first rib remains the treatment of choice for patients who have recurrence of symptoms.^[7,8]^ Indeed, some studies have reported that 40% of patients who do not undergo surgical decompression will eventually need surgery for recurrent symptoms, and only 63% of patients will have symptom-free status at last follow-up.^[1]^ However, early surgical intervention still remains controversial. Occasionally, transaxillary surgical results are hampered because surgical inexperience leads to insufficient rib removed. As in the case of the 2nd patient, inadequate amounts of the anterior portion of the rib was not sufficiently removed, leading to persistent compression.

In general however, the Transaxillary approach has advantages because it allows for excellent exposure of the anterior and posterior portions of the first rib through a small axillary incision.^[7,8]^ Stent placement can help to ameliorate symptoms, but it can be challenging if there is continued extrinsic compression of the vein, as our 2nd case demonstrated, in which the residual first rib compressed the patient's stent. In addition, the longevity of such venous stents remains unknown and the concern for stent fracture may lead to a recurrence of venous thrombosis; particularly since patients who are afflicted with VTOS are mostly young patients. Hence, we do not routinely recommend venous stenting in patients with VTOS. Instead, we usually employ angioplasty followed by a limited period of anticoagulation.^[9]^ This is of benefit to active patients who may be restricted from certain activities, particularly contact sports, when they are on anticoagulation.

As evident in the 2 cases described above, UEDVTs can be debilitating and may limit activities of daily living. Surgical decompression may offer significant improvement in quality of life and symptom relief in such patients.

## 5 Author contributions

**Writing – original draft:** M. Yunce, Y.W. Lum.

**Writing – review & editing:** M. Yunce, A. Sharma, E. Braunstein, M.B. Streiff, Y.W. Lum.
